# Theranostic Role of Iron Oxide Nanoparticle for Treating Renal Anemia: Evidence of Efficacy and Significance by MRI, Histology and Biomarkers

**DOI:** 10.3390/pharmaceutics15061714

**Published:** 2023-06-12

**Authors:** Jong-Kai Hsiao, Chih-Lung Chen, Wen-Yuan Hsieh, Ko-Lin Kuo

**Affiliations:** 1 Department of Medical Imaging, Taipei Tzu Chi Hospital, Buddhist Tzu-Chi Medical Foundation, New Taipei City 23142, Taiwan; 2 School of Medicine, Tzu Chi University, Hualien 97004, Taiwan; 3 Division of Translational Medicine, MegaPro, Ltd., Hsinchu 30204, Taiwan; 4 Division of Nephrology, Taipei Tzu Chi Hospital, Buddhist Tzu Chi Medical Foundation, New Taipei City 23142, Taiwan; 5 School of Post-Baccalaureate Chinese Medicine, Tzu Chi University, Hualien 97048, Taiwan

**Keywords:** chronic kidney disease, cytokines, iron deficiency anemia, iron oxide nanoparticles, oxidative stress, magnetic resonance imaging

## Abstract

(1) Background: Increasing attention has been given to applying nanosized iron oxide nanoparticles (IOPs) to treat iron deficiency anemia (IDA). Chronic kidney disease (CKD) patients who suffer from IDA often need long-term iron supplements. We aim to evaluate the safety and therapeutic effect of MPB-1523, a novel IOPs, in anemic CKD mice and to monitor iron storage by magnetic resonance (MR) imaging. (2) Methods: MPB-1523 was intraperitoneally delivered to the CKD and sham mice, and blood were collected for hematocrit, iron storage, cytokine assays, and MR imaging throughout the study. (3) Results: The hematocrit levels of CKD and sham mice dropped initially but increased gradually to reach a steady value 60 days after IOP injection. The body iron storage indicator, ferritin gradually rose and total iron-binding capacity stabilized 30 days after IOP injection. No significant inflammation or oxidative stress were observed in both groups. By T2-weighted MR imaging, the liver signal intensity gradually increased in both groups but was more pronounced in the CKD group, indicating aggressive utilization of MPB-1523. MR imaging, histology and electron microscopy showed MPB-1523 is liver-specific. (4) Conclusions: MPB-1523 can serve as a long-term iron supplement and is monitored by MR imaging. Our results have strong translatability to the clinic.

## 1. Introduction

Anemia affects one-third of the global population, and iron deficiency anemia (IDA) is its leading cause [1,2]. Oral iron supplementation and intravenous (IV) iron infusion are two major treatment strategies [3]. However, the recovery of IDA by oral iron supplementation is very slow. Additionally, adverse reactions to oral iron supplements, such as nausea, vomiting, and alteration of taste, often hinder treatment compliance [1]. IV iron infusion, such as iron sucrose, provides fast delivery of sufficient iron to the human body, but the allergic reaction that can occur immediately after infusion hinders its application. Recently, ultrasmall superparamagnetic iron oxide (USPIO), named ferumoxytol, has been used in clinical applications for oral iron-resistant IDA treatment. The hydrodynamic diameter of ferumoxytol is 30 nm and it has an iron oxide core and low molecular weight carbohydrate coating [4]. However, the slow infusion of ferumoxytol and its considerable serious adverse effects limit its clinical usage [5].

Anemia is more common in patients with chronic kidney disease (CKD). The causes of CKD-associated anemia are complex. Erythropoiesis is mainly suppressed due to inadequate kidney erythropoietin (EPO) production. Other factors of renal anemia include absolute or relative iron deficiency, inflammation, uremic toxin-induced inhibition of erythropoiesis, nutritional deficiencies of folate and vitamin B12, and shortened red blood cell survival [6]. In addition to insufficient EPO production, iron deficiency plays a critical role in CKD-associated anemia. [6]. CKD patients tend to have a higher risk of iron loss because of chronic bleeding from platelet dysfunction, frequent phlebotomy, and blood trapping in the hemodialysis (HD) apparatus. Moreover, CKD patients also have impaired dietary iron absorption, making oral iron supplements less effective than IV iron supplementation regarding improving iron deficiency and reducing EPO dose in HD patients [7,8,9]. Therefore, IV iron is preferred for HD patients because of impaired dietary iron absorption. However, according to our previous studies, parental iron supplementation is prone to producing labile iron, which generates oxidative stress and is then associated with adverse clinical prognosis in CKD [10,11,12]. Therefore, developing a novel iron species to reduce potential side effects while maintaining anti-anemic efficacy is an essential and important issue in the treatment of CKD anemia.

Unlike USPIO which is defined as nanoparticle diameter less than 50 nm [13,14], superparamagnetic iron oxide (SPIO) nanoparticles have a larger iron oxide core more than 50 nm, providing a greater iron capacity for single-shot treatment. It has been known that iron oxide nanoparticle (IOP) less than 20 nm will be eliminated by the kidneys whereas IOP larger than 200 nm would be uptake by macrophages located in the splenic marginal zone red pulp [13]. Only IOPs at the range between 20 nm and 200 nm could be uptake by the liver Kupffer cells where adjacent hepatocytes could convert the iron oxide into a ferrous component of ferritin that could be further transformed into hemoglobin in the red blood cells. Although the size of IOP is critical in the treatment of anemia, the particle coating, surface charge and size all contribute to the biodistribution of the IOP that is influential to the renal anemia therapeutic effect. It has been known that polyethylene glycol coated IOPs have higher blood half-life [15]. The surface charged IOP are prone to bind plasma proteins or cells, leading to faster elimination in the plasma [16]. Consequently, IOPs that are designed for treating anemia are composed of negatively changed polymers such as a polyglucose sorbitol carboxymethyl ether coating in ferumoxytol [17].

Among the various size of IOPs, SPIO has been widely used for liver magnetic resonance imaging (MRI) over the past 20 years [18]. SPIO particles are very safe and are composed of biodegradable/bioavailable iron, which is biocompatible and can thus be reused or recycled by cells using normal biochemical pathways for iron metabolism. Ferumoxides (Feridex^®^ and Endorem^®^) and ferucarbotran (Resovist^®^) are commercially available as SPIO contrast agents. They are IV injected and allow a shortened examination time, dynamic perfusion, and better and more specific contrast enhancement when performing MRI.

MegaPro Biomedical has developed a nanosized iron oxide particle (IOP) injection (MPB-1523) as a safe MRI contrast agent for metastases or immune cell tracking [19,20,21]. Injected IOPs coated with biocompatible/biodegradable m-PEG-silane can be easily phagocytosed by reticuloendothelial system (RES) cells. IOP injection can very effectively shorten the T2 relaxation time and reduce signal intensity in normal liver tissues. Furthermore, MPB-1523 shows a better imaging effect than the commercially available product Resovist^®^ in vivo. MPB-1523 has passed a phase 2 clinical trial, proving its safety and efficacy. Consequently, we propose that MPB-1523 has a therapeutic effect on IDA and could be imaged by clinical MRI in a CKD animal model.

Therefore, we first performed subtotal nephrectomy to mimic clinical CKD status and induce renal anemia. Then, iron deficiency status was accomplished by blood sampling using phlebotomy in the first three days in both the sham and CKD groups. By the novel experimental design, this study primarily aims to evaluate the safety, tolerance, and therapeutic effect of MPB-1523 in CKD mice with anemia. The 2nd aim is to monitor iron storage noninvasively by MRI and correlate it with histological findings at the study endpoint.

## 2. Materials and Methods

### 2.1. Reagents

IOP (MPB-1523) was provided by MegaPro Biomedical (Zhubei, Taiwan). MPB-1523 is a water-soluble SPIO nanoparticle chemically conjugated with m-PEG-silane. It is administered via IOP injection as a sterile, aqueous colloidal solution with 20 mg/mL elemental iron and particle sizes ranging from 45–65 nm. TIBC and serum iron assay kits, enzyme-linked immunosorbent assay kits and tissue staining reagents were obtained from Abcam (Cambridge, UK).

### 2.2. Animal Procedures

**Figure 1:**
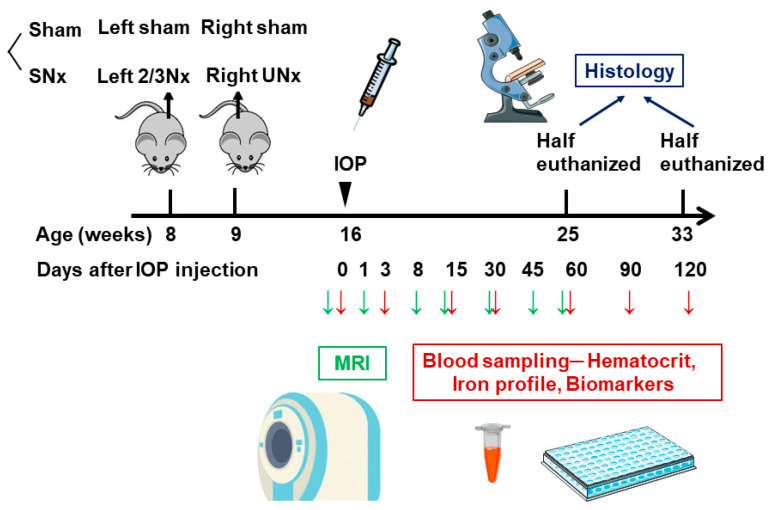
Study protocol of IOP injection in sham and SNx mice. Mice were divided into 2 groups including sham operation (Sham) treated with IOP, and subtotal nephrectomy (SNx) treated with IOP. *N* = 8 in sham group and *N* = 12 in SNx group IOP, iron oxide nanoparticle; SNx, subtotal nephrectomy; 2/3 Nx, 2/3 nephrectomy; UNx, uninephrectomy.

All experimental procedures and protocols involving animals were carried out in accordance with the institutional animal care committee of Taipei Tzu Chi Hospital and complied with the Guide for the Care and Use of Laboratory Animals (IACUC approval number: 109-IACUC-008). Twenty male C57BL/6 mice were divided into two groups, with 12 mice undergoing subtotal nephrectomy (SNx) and 8 mice undergoing sham operation. SNx surgery was induced in 8-week-old mice using a 2-step surgical nephrectomy procedure as previously described [12,22,23]. Briefly, mice were anesthetized, and the left kidney was exposed through an incision at the dorsal midline skin. Then, two of three branches of the left renal artery were ligated through a lateral incision. One week after the operation, the right kidney was removed after ligation of the renal blood vessels and ureter under anesthesia. Previous research suggests that using an iron sucrose-loaded rodent model through intraperitoneal administration can provide results that are comparable to those obtained through intravenous administration in humans [24]. Additionally, due to the small size of the tail veins in mice, repeated intravenous injections can be difficult to administer. We chose intraperitoneal IOP administration. According to the report of the previous phase 2 study, the recommended human dosage for IOP (MPB-1523) is 6 mg/kg. By applying the formula to determine the human equivalent dose for mice, this would translate to approximately 1.8 mg for a 25 g mouse. Therefore, each mouse was intraperitoneally administered a single dose of IOP at a volume of 90 µL, equivalent to 1.8 mg iron per kg of body weight. This administration was carried out seven weeks after SNx. Then, approximately 0.3 mL of blood was collected from the submandibular vein at 0, 3, 14, 30, 60, 90, and 120 days after IOP injection. Each blood sample was separated into a K_2_ EDTA tube for hematocrit measurement and a serum tube for iron parameter and ELISA measurements. The study protocol of IOP injection in sham and SNx mice was summarized in Figure 1.

### 2.3. Hematocrit Measurements

Whole blood was collected in a K2 EDTA tube and mixed thoroughly by inverting it gently. A capillary tube was placed in the blood sample, and blood was drawn into the tube until the tube was approximately 2/3 full. After sealing the end that was placed in the sample by pressing it into the clay, the capillary tube was centrifuged at 10,000× *g* for 5 min with Micro hematocrit centrifuge Kubota 3320 (Osaka, Japan). Hematocrit was immediately evaluated by calculating the ratio of the column of packed erythrocytes to the total length of the sample in the capillary tube, as measured with a graphic reading device.

### 2.4. Iron Parameters

Serum was separated by centrifugation at 2000× *g* for 15 min and stored at −80 °C before use. Serum iron, transferrin saturation (TSAT), and total iron-binding capacity (TIBC) were determined using TIBC and serum iron assay kits (Abcam, ab83366, Cambridge, UK) according to the manufacturer’s instructions. Sensitivity of the kit was 8 µM.

### 2.5. ELISA

Separated animal serum was stored at −80 °C before measurement. The ferritin, IL-6 and TNF-α concentrations in serum were determined using an enzyme-linked immunosorbent assay kit (Abcam, ab157713, ab222503, ab208348, Cambridge, UK) according to the manufacturer’s instructions.

### 2.6. Histology

Organs harvested from the sacrificed mice were fixed with 4% paraformaldehyde, embedded in paraffin and serially sectioned at 5 mm. Slides generated from the sections were subjected to hematoxylin and eosin (HE) staining. Iron deposits were examined using Pearls’ Prussian blue reaction. The slides were the incubated with 4% potassium ferrocyanide in 4% hydrochloric acid for 10 min and then counterstained with a fast red substrate kit (Abcam) for 5 min. Histological images were photomicrographed under a Nikon ECLIPSE 80i microscope (Tokyo, Japan) with QCapture Pro 6.0 software. The samples for transmission electron microscopy (TEM) images were sectioned at a thickness of 100-nm on a Reichert-Jung Ultracut-E ultramicrotome. The sections were viewed on a Hitachi 7100 transmission electron microscope (Tokyo, Japan) at 50 keV. Images were recorded with an AMT Advantage 10 Image Acquisition System using a Kodak Megaplus 1.6i CCD camera system (1024 × 1024 TIFF format) [19].

### 2.7. MRI

Mice that underwent SNx for four weeks were administered IOP (MPB-1523) via intravenous injection. These mice were then imaged with a 7T-MRI system (Biospec 70/30; Bruker, Billerica, MA, USA). RARE-T2 pulse sequences provided by the vendor were used (TR/TE = 5000/56 ms; flip angle = 180°, matrix size = 256 × 256). The slice thickness was 1.0 mm, and the field of view was 6 × 4 cm, with coverage of the abdominal cavity from the diaphragm to the pelvic floor. The total scan time was 3 min and 20 s at a number of excitations (NEX) of 3. The images were analyzed with ImageJ software.

### 2.8. Immunohistochemistry

Mice liver sections were deparaffinized, rehydrated, and incubated with 3% H_2_O_2_ for 10 min. After blocking with 1% BSA for 1 h, the samples were incubated with anti-ferritin (abcam, ab287968, Cambridge, UK) or anti-transferrin (abcam, ab214039, Cambridge, UK) antibody overnight at 4 °C and then incubated with a fluorescein isothiocyanate-conjugated secondary antibody for 2 h. Immunoreactions were visualized under a TE2000-U fluorescence microscope (Nikon, Melville, NY, USA).

### 2.9. Statistical Analysis

All data are presented as the mean ± SEM values from five to ten independent experiments. The Mann–Whitney test was used for comparisons between two independent groups. SPSS software version 18.0 (IBM, Armonk, NY, USA) was used for statistical analysis. Differences were considered statistically significant at *p* < 0.05.

## 3. Results

### 3.1. The Chemical Properties and Stability of IOP Injection

MPB-1523 (IOP Injection), developed by Megapro Biomedical Ltd., is an SPIO nanoparticle chemically conjugated with m-PEG-silane that is soluble in water. IOP injection is a sterile, aqueous, colloidal solution with 20 mg/mL elemental iron and particle sizes ranging from 45–65 nm in diameter. The formulation is isotonic with an osmolality of 260–340 mOsm/L and a pH range from 5.5 to 7.5 (Table 1).

### 3.2. Changes in Hematopoiesis in Sham and SNx Mice after IOP Injection

**Figure 2:**
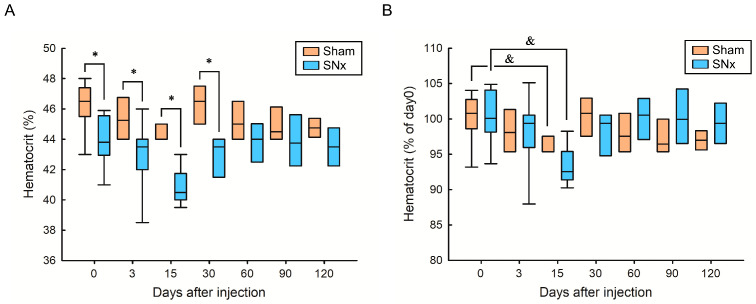
Effect of IOP injection on hematocrit in sham and SNx mice, (**A**) comparing its impact between the two groups and (**B**) assessing the time-dependent effect. * *p* < 0.05 compared with respective period of sham group. ^&^ *p* < 0.05 compared with its baseline level.

Our study protocol of IOP injection in sham and SNx mice was summarized in Figure 1. This study first performed subtotal nephrectomy to mimic clinical CKD status and induce renal anemia in the SNx group. Aggressive blood sampling by phlebotomy was performed in the first three days to aggravate the iron deficiency status in both the sham and SNx groups. Therefore, we could easily compare the effects of hematopoiesis after a single dose of IOP injection in sham and SNx mice throughout the study. We first found that compared with the sham group, the SNx group had significantly lower mean hematocrit levels on Day 0, Day 3, Day 15, and Day 30. The difference between the sham and SNx groups diminished from Day 60 to Day 120 (Figure 2A). The mean hematocrit level was 45.3% on Day 3 and 44.5% on Day 15 in the sham group (data are expressed as the mean ± SEM). The mean hematocrit level was 43.1% on Day 3 and 40.9% on Day 15 in the SNx group. The mean hematocrit levels in both groups were significantly lower than baseline on Day 15, gradually increased on Day 30 and remained stable afterward (Figure 2B).

### 3.3. Changes in the Iron Parameters in Sham and SNx Mice after IOP Injection

**Figure 3:**
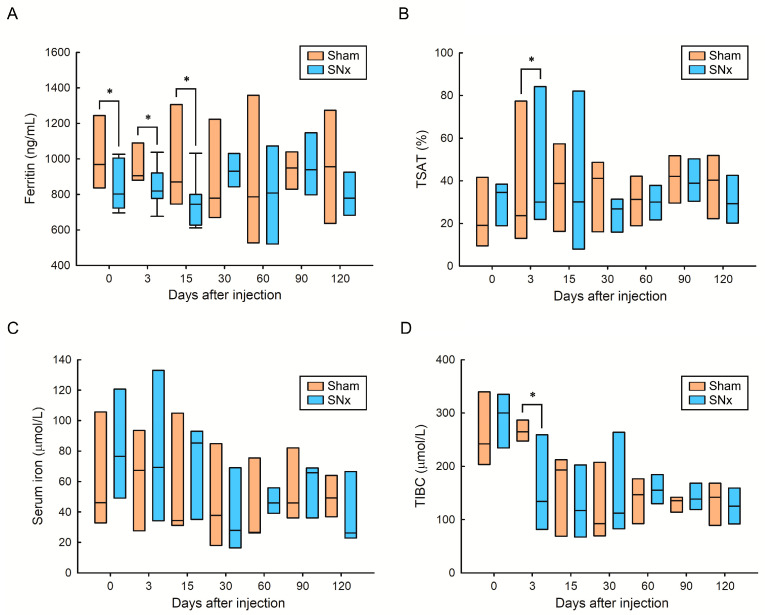
Effect of IOP injection on serum ferritin (**A**), TSAT (**B**), serum iron (**C**) and TIBC (**D**) in sham and SNx mice throughout the study. * *p* < 0.05 compared with respective period of sham group.

To evaluate the effects of IOP injection on iron parameters in both groups, we measured serum ferritin, TSAT, serum iron, and TIBC levels in the sham and SNx mouse groups after IOP injection (Figure 3). Compared with the respective period of the sham group, the serum ferritin level (an index of iron storage) in the SNx group was significantly lower on Day 0, Day 3, and Day 15. The difference between the sham and SNx groups diminished from Day 60 to Day 120 (Figure 3A). In contrast, the circulating TSAT level (an index of circulating iron) in the SNx group was significantly lower on Day 3 than that in the sham group during the corresponding period (Figure 3B). This indicates that a single dose of IOP injection could increase circulating iron on Day 3 and then recover stable iron storage in CKD mice at Day 30. Finally, we found that the mean serum iron and TIBC levels in the SNx group were not significantly different compared with those in the sham group of mice during the same time period, except the TSAT level on Day 3 (Figure 3C,D).

### 3.4. Changes in Proinflammatory Cytokines and Oxidative Stress in Sham and SNx Mice after IOP Injection

**Figure 4:**
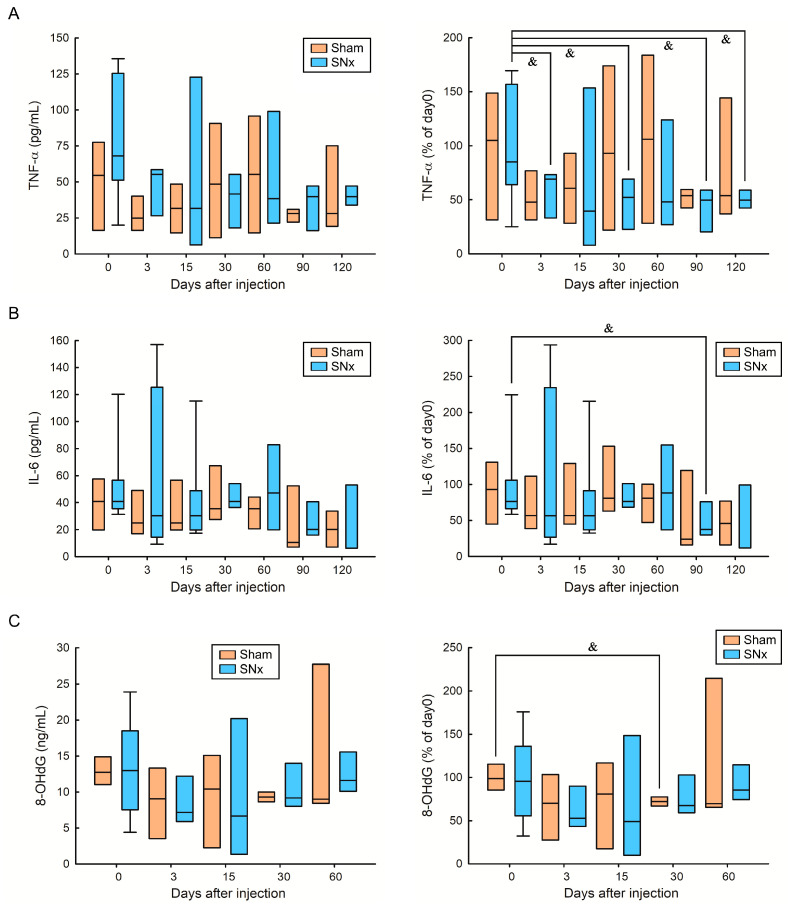
Effect of IOP injection on serum TNF-α (**A**), IL-6 (**B**), serum 8-OHdG (**C**) in sham and SNx mice throughout the study. ^&^ *p* < 0.05 compared with its baseline level.

To evaluate the effect of IOP injection on proinflammatory status and oxidative stress, we measured serum TNF-α, IL-6, and serum 8-OHdG in the sham and SNx mouse groups after IOP injection (Figure 4). We first observed that the mean serum levels of TNF-α, IL-6, and 8-OHdG were not significantly different between the sham and SNx mouse groups throughout the study. In Figure 4A, we found that the trend of serum TNF-α in the sham group decreased throughout the study compared with the baseline, but the values did not achieve statistical significance. Moreover, the serum TNF-α level was significantly decreased on Days 3, 30, 90, and 120 compared with the baseline level in the SNx groups. In Figure 4B, we found that the changes in serum IL-6 between the sham and SNx groups did not achieve statistical significance compared with their baseline levels. In addition, only the serum TNF-α level significantly decreased on Day 90 compared with the baseline level in the SNx groups. Finally, we found that the changes in serum 8-OHdG between the sham and SNx groups did not achieve statistical significance compared with their baseline levels (Figure 4C). Only the serum 8-OHdG level significantly decreased on Day 30 compared with the baseline level in the sham group.

### 3.5. MRI Visualization of the Abdomen before and after IOP Injection

**Figure 5:**
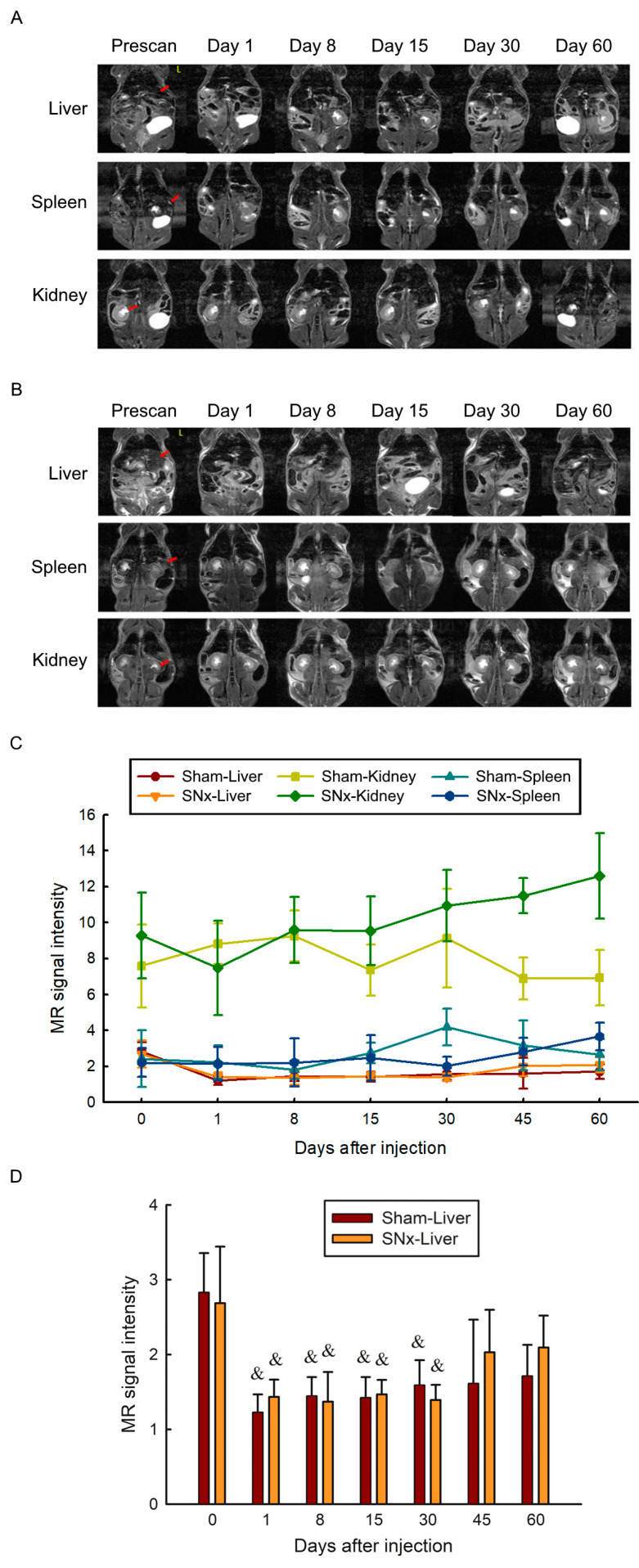
Serial T2 weighted MRI study of the SNx mice (**A**) and sham control mice (**B**) before and after IOP administration. MR images of three organs including liver, spleen and kidneys were illustrated. There is no visible organ damage after IOP administration. Significant liver signal intensity drop is observed after IOP administration. Quantitative measurement of the liver and renal signal intensity was illustrated (**C**). After the single dose of IOP delivery, the signal intensity of the liver elevated gradually which is more pronounced in the SNx group. There is no significant signal intensity change of the spleen in both groups. (**D**) Quantification of MR signal intensity of liver before and after single dose IOP injection in sham and SNx mice. ^&^ *p* < 0.05 compared with its baseline level before IOP injection. Data are expressed as mean ± SEMs.

To identify IOP storage sites in the abdominal cavity, serial T2-weighted magnetic resonance images were acquired at intervals between 7 and 14 days. There was a significant signal intensity drop in the liver in both sham control and SNx mice immediately after IOP injection on the first day. The signal intensity of the liver increased gradually at the observation endpoint (Day 60). There was more pronounced liver signal elevation in the SNx group than in the sham control group. A steady, slowly signal intensity increase was observed in the spleen in both the sham control and SNx mice (Figure 5).

### 3.6. Histological Analysis of the Presence of IOPs

**Figure 6:**
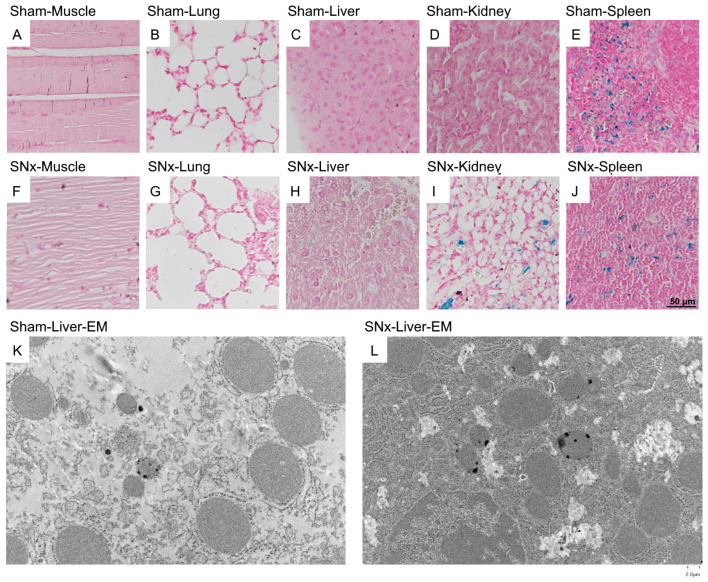
Histological observation of organs including skeletal muscle, lung, liver, kidney, spleen and residual kidney of SNx group for the existence of IOP by Pearl Stain with counter stained with Acid Fast (**A**–**J**) and Electron microscopy of liver of sham control (**K**) and SNx group (**L**). The pearl stain showed blue staining at the trabecula region of spleen and inside the vasculature of the liver in both sham control and SNx group. The residual kidney of the SNx group (**I**) had significant pearl staining. EM of the liver in both sham control and SNx group showed presence of small amount of IOP inside the vesicle organelle of the hepatocyte.

To identify IOP injection metabolism in SNx mice, specimens of skeletal muscle, lung, liver, kidney, and spleen from both sham control and SNx group mice were stained with Pearl’s stain (Figure 6A–J). The blue staining representing the presence of iron could be visualized at the splenic trabecula of both the sham and SNx groups. The liver hepatocytes did not show Pearl staining. Instead, the Kupffer cells of the sham control group showed blue iron staining (Figure 6C). There was significant blue staining in the kidney that had undergone surgery in the SNx group (Figure 6I). In addition, no histological changes in the organs were observed. We further studied the presence of IOPs by electron microscopy. Under 15,000× magnification, a trace number of black dots could be visualized at the vesicular-bound organelles of the liver in both the sham control and SNx groups. In addition, we also found pronounced Pearl staining in the sham control group in the liver and spleen (Figure 6C,E) compared with the SNx group (Figure 6J).

### 3.7. Ferritin and Transferrin Expression in Liver

**Figure 7:**
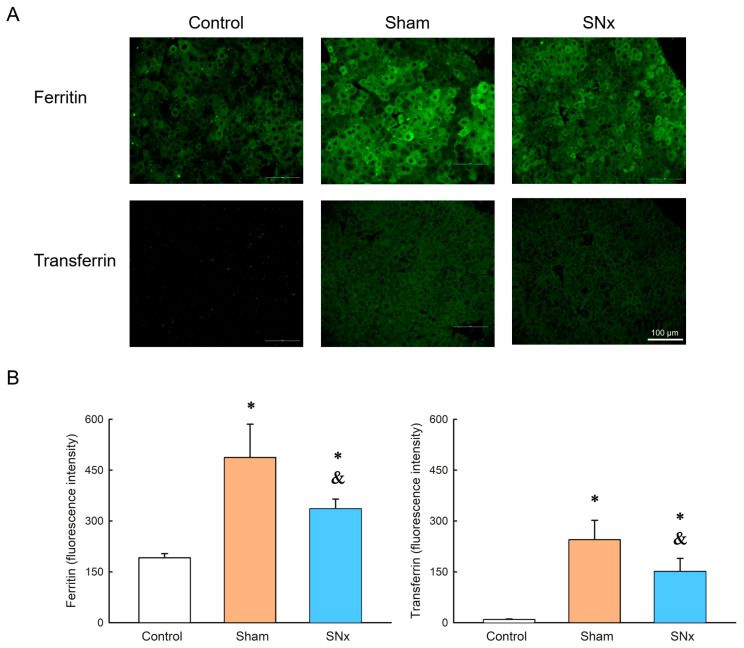
Representative images (**A**) and quantification (**B**) of immunohistochemistry of liver tissues from control, SNx and sham groups 60 days after IOPs delivery. Ferritin expression could be observed in both SNx and sham groups and there is stronger ferritin expression in sham group as compared to SNx group. Moreover, the transferrin expression was similar as the expression of ferritin in these three groups. * *p* < 0.05 compared with control group. ^&^ *p* < 0.05 compared with its sham group. Data are expressed as mean ± SEMs.

Immunohistochemistry of liver tissue obtained from control, sham and SNx groups at the endpoint of the MRI study showed there is ferritin expression in SNx and sham group, in which the sham group had stronger ferritin expression. Meanwhile, the transferrin expression was similar as the expression of ferritin in these three groups. (Figure 7).

## 4. Discussion

We established a CKD/IDA combined mouse model to verify the IOP treatment effect. There was a steady increase in hematocrit after IOP injection. Iron storage is also restored without interference from proinflammatory cytokines. Moreover, this polyethylene glycol (PEG)-coated IOP was very liver-specific and could be steadily metabolized, making it an ideal agent to treat IDA and a tool for MR imaging of the liver iron reserve.

Iron has crucial roles in supporting physiological functions, such as oxygen transport through hemoglobin, which an essential process associated with respiration, and DNA synthesis and repair [25]. However, iron is a cellular transition element, and its ionic forms are prone to participate in one-electron transfer reactions. This property also allows iron to generate reactive oxygen species (ROS). In the presence of iron, reactive hydroxyl radicals can be formed via the Fenton reaction and the iron-catalyzed Haber-Weiss reaction [26,27]. The generated ROS further trigger several types of damage to the human body. Therefore, while facing treatment of IDA or iron deficiency in CKD, which is also a status of high oxidative stress, the adverse effects of iron supplementation should be considered and balanced with its therapeutic benefits [10,11,12,28,29,30]. Current iron therapies suffer from a few drawbacks. First, they have low potency and require high dosage or frequent dosing. Second, they have adverse side effects, such as anaphylaxis and hypersensitivity. Indeed, patient compliance is low due to their adverse effects. There is a need to develop a potent and safe parenteral iron therapy [25].

In managing IDA, oral iron therapy is a simple, inexpensive treatment but is limited by gastrointestinal side effects, poor adherence, and impaired absorption in some patients, while it is also of minimal efficacy in others [28]. On the other hand, IV iron is more effective than oral iron supplementation for replenishing iron stores, improving anemia, and reducing ESA dosage requirements in CKD [28]. However, the cumulative release of labile iron during IV iron infusion might further increase the risk of oxidative stress accumulation and further induce endothelial dysfunction, inflammation, and even cardiovascular events or bacterial infection [11,25]. Therefore, ideal IV iron preparations should have low adverse effects, ease of administration, and low free-iron toxicity [20]. Recently, novel iron products such as ferumoxytol, ferric carboxymaltose, and iron isomaltoside can bind iron more tightly, which minimizes the release of labile iron during IV infusion, is associated with better patient tolerance and were shown to be noninferior to established iron formulations. However, it remains to be determined whether they are superior to traditional parenteral supplementation or oral iron products with respect to long-term risks and benefits [31].

Traditional iron preparations are often bioengineered as iron-carbohydrate complexes to deliver iron in a stable, nontoxic matter and consist of colloidal suspensions of iron oxide nanoparticles with a polynuclear Fe(III)-oxyhydroxide/oxide core surrounded by a carbohydrate ligand [25,32,33]. The surrounding carbohydrates play a pivotal role in in vivo iron release and maintain colloidal suspension of the IOPs [34,35]. After parenteral administration, the complexed iron is taken up by phagocytic cells in the RES and then merged into ferritin. These iron-containing complexes could be stored intracellularly or bound to transferrin and further delivered as erythroid precursors for hemoglobin synthesis. However, the newly synthesized IOP MPB-1523 is coated with PEG rather than carbohydrate ligand. In addition, the central core of the nanoparticle consists of Fe_3_O_4_ [19,20,21]. From a clinical viewpoint, several issues merit discussion in this study. First, this novel design could provide high iron dosage loading with slow iron release and a lower risk of anaphylaxis and hypersensitivity. Theoretically, these factors could increase patient compliance and safety in clinical IDA treatment. Second, from a pharmacokinetics view, we found that a single IOP injection could produce an earlier increase in circulating iron (indexed by TSAT level) beginning on day three and restore stable iron storage (indexed by serum ferritin level) in CKD mice at Day 30. Moreover, the hematocrit level gradually increased after Day 30 and remained stable throughout the study. Third, CKD presents a proinflammatory status and is associated with high oxidative stress [36]; a high dose of parenteral iron could further induce more oxidative damage [10,11,12]. Moreover, intraperitoneal IOP administration did not change the oxidative status in CKD mice. Surprisingly, our study revealed that IP IOP administration could decrease the proinflammatory cytokines TNF and IL-6 rather than exacerbate the inflammatory response in CKD mice. We propose the surface coating of PEG contribute to the inner immune property. As porcine model showed noticeable allergic reaction after ferumoxyltol treatment [37]. Our next step in further studies will be to evaluate the efficacy and potentiation of allergy between the MegaPro and ferumoxytol since both are IOPs with different coatings. The safety of IOPs is well demonstrated in our study. Morphological research also showed intact livers and kidneys 6 months after IOP delivery. Our study showed persistent low MR signal intensity in the liver, but Pearl staining revealed no iron oxide nanoparticles. However, a trace amount of IOP particles were found in the TEM study. The findings are evidence of physiological metabolization of IOPs into body iron storage forms, such as ferritin or hemoglobin. Similar findings were reported in a mouse model in which 70 µg of iron oxide nanoparticles were injected intravenously, and serial transmission electron microscopy showed progressive degradation of the particles. An in vivo study showed that the liver reserve of iron increased after the IV IOP injection and returned to standard levels 180 days later [38]. We found that MR images are a powerful in vivo tool for evaluating iron reserves. The hematocrit level in CKD mice was maintained 120 days after frequent blood sampling. We also noted that the disappearance of the IOPs in the TEM study is not representative of the iron reserve, which is crucial in renal anemia. Although blood sampling could reveal several aspects of body iron reserve, frequent blood sampling in small animals might significantly impact blood loss. Therefore, MRI observation of the liver as a tool for body iron reserve is more physiologically meaningful.

IOPs are metabolized in the blood compartment, especially Kupffer’s cells, rather than in the hepatic compartment, such as hepatocytes [39,40]. IOPs transform into ferritin in hepatocytes [41]. Our study showed a constantly low signal intensity in the liver by MRI, indicating the persistence of iron metabolism in the liver. This finding was further proven by TEM. There was less Perl’s staining in the CKD liver than in the sham control livers, which is another evidence of iron oxide metabolization. Furthermore, it is known that delivered IOPs are metabolized in liver where ferritin is the main protein responsible for iron storage [42]. In our study, both the serum ferritin level and liver IHC of ferritin indicated increased ferritin expression in the sham group compared with the CKD group, which means the delivered IOPs are efficiently metabolized. We propose that IOP is actively metabolized into hemoglobin, which alleviated anemia in the CKD group. MRI could be considered a nondestructive measurement of the iron pool of the liver, which is crucial in the treatment of anemia in CKD patients. Moreover, the signal intensity change is not observed in the spleen, which lymphocytes predominantly occupy. This finding is also consistent with the histological findings. Similar results have been observed in clinical MR IOPs, such as Ferucarbotran [43]. The long-term therapeutic effect of IOPs and IOPs’ inherent MRI detectability makes this IOPs an ideal theranostic agent for treating renal anemia.

There are limitations of this study. First, our study did not show the comparisons of therapeutic effect and safety of IP IOP with conventional drugs, such as iron sucrose. However, the current study is to demonstrate the feasibility of monitoring iron storage status by MRI with concurrent observation of hemoglobin recovery. The comparison study would be our next study focus. Second, our experiment showed the IP IOP administration could decrease the proinflammatory cytokines of TNF and IL-6 and oxidative stress, but includes no data demonstrating the lower risk of anaphylaxis and hypersensitivity. Third, although we did semi-quantative T2 weighted imaging serially, the T2 map were not acquired which would enhance the evidence of the study. Future work on the comparison between ferumoxytol and MPB-1523 with T2 map should be performed. Fourth, iron can be quantified with greater sensitivity (at parts per trillion, ppt) with, for example, the ICP-MS technique, but we did not employ such techniques in this study. Since our study design is a pilot study in nature, more animal and human study needs proof our results in the future.

## 5. Conclusions

The high safety, single-dose delivery, and long duration of new IOPs for treating iron deficiency in CKD patients have been well demonstrated. Moreover, IOPs are very liver specific, making them an ideal tool for MR liver imaging to detect liver tumors in cancer patients and a monitoring agent for iron storage conditions. The IOP in this study has finished its phase 2 clinical trials, which could accelerate its translatability into the clinic. Owing to the good tolerance of the IOP in our animal study, we believe that new IOPs could change the landscape of CKD-related IDA and other IDA induced by chronic disease.

## Figures and Tables

**Table 1:** Stability data for IOP injection.

**Item**	**Acceptance Criteria**	**IOP Injection (Batch No.: MPB-1523)**	
		**Condition: 5 ± 3 °C**	
		**Testing Interval, Month**	
		**0**	**12**
Appearance	Dark brown to black solution	Confirm	Confirm
Identification	Corresponds to the reference peaks	Confirm	Confirm
Assay	20.0 ± 2.0 mg/mL	20.6 mg/mL	20.2 mg/mL
Particle size	Z average: 65 ± 20 nm; (PDI < 0.3)	60 nm; PDI = 0.2	61 nm; PDI = 0.2
Zeta potential	0~−20 mV	−2 mV	−2 mV
Bacterial endotoxin	NMT 6 EU/mL	<6 EU/mL	<6 EU/mL
Sterility	Sterile	Sterile	Sterile
pH	5.5–7.5	6.3	5.7
Impurity	Individual impurity: <0.5% Total impurities: <3.0%	Individual impurity: ND Total impurities: ND ^1^	rrt 1.00: 0.4% rrt 1.20: 0.4% rrt 1.24: 0.4% Total impurities: 1.3%

^1^ GPC method-mobile phase: H_2_O, column: SB803 + SB802.5 column, detector: RI. rrt: relative retention time (RT of unknown impurity/RT of mPEG 2000).

## Data Availability

Not applicable.
